# Microfabrication Technologies for Interaction Circuits of THz Vacuum Electronic Devices

**DOI:** 10.3390/mi15111357

**Published:** 2024-11-08

**Authors:** Xinghui Li, Jinjun Feng

**Affiliations:** National Key Laboratory of Science and Technology on Vacuum Electronics, Beijing Vacuum Electronics Research Institute, Beijing 100015, China; fengjinjun@tsinghua.org.cn

**Keywords:** THz, vacuum electronics device, interaction circuit, FWG, microfabrication, LIGA/UV-LIGA, DRIE, CNC-milling, 3D printing

## Abstract

Advances in manufacturing technology are allowing for the realization of interaction circuit with microstructures. The capability to produce small circuit structures is allowing new opportunities for vacuum electronic devices producing terahertz (THz) frequency radiation, which is impractical with traditional machining technology. This publication reviews recent progress on advanced microfabrication technologies applicable to interaction circuits of THz vacuum electronic devices, including LIGA/UV-LIGA (Ultraviolet Lithographic, Galvonoformung and Abformung), deep reactive ion etching (DRIE), micro/nano computer numerical control (CNC) milling, three-dimension (3D) printing, etc., and describes the current State-of-the-Art of their applications.

## 1. Introduction

Terahertz (THz) is in the electromagnetic spectrum located between the infrared and microwave wave bands, and has historically been referred to by different frequency ranges in different literature [1,2,3]. For expediency, THz regime, in this paper, is defined as the frequency range of 0.1–10 THz, even though a more accurate and elaborate terminology might include millimeter-wave, submillimeter-wave, and THz [4].

The shorter wavelengths at THz frequencies allow the use of smaller and lighter components, which is especially important in military and space-borne applications where size and weight are a prime concern. Therefore, THz technology has numerous potential application fields, including high-data-rate communications [5], high-resolution radar and imaging [6,7], medicine and biology [8], and astronomy [9].

However, THz band of frequency 0.1–10 THz is still one of the least explored frequency bands, and the critical barrier to fully exploiting the THz band is attributed mainly to the lack of compact, powerful, and convenient THz sources operating at room temperature [10].

Many THz sources were proposed and developed during the past decades to change this situation, including quantum cascade lasers, optically pumped solid state devices, semiconductor solid state devices (SSDs), and free electron-based vacuum electronic devices (VEDs) [11,12]. The THz radiation generation mechanism of these sources is the extension of microwave electronics towards high frequencies on one side, or the frequency down-conversion from the optical region on the other side.

SSDs and VEDs are two main types of electronic radiation sources dominating lower-frequency THz applications. Solid-state sources have the advantages of compactness and integrability, thanks to their mature manufacturing technology. However, due to the physical limits on power density management, vacuum electronic sources present better practical solutions for high power, efficient sources of 0.1–1.0 THz radiation [13].

**Figure 1:**
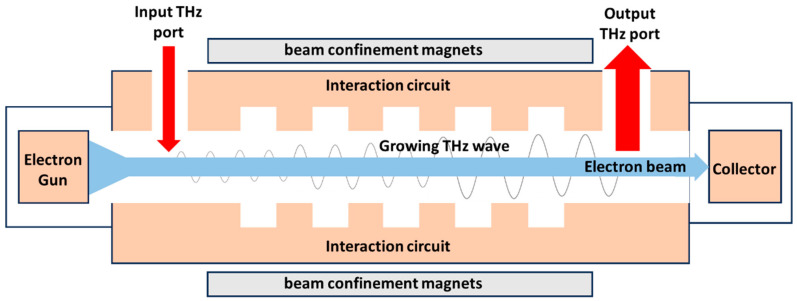
Conceptual illustration of a THz vacuum electron amplifier (oscillator).

Oscillators and amplifiers are the two main VEDs terahertz sources; the conceptual illustration of them is shown in Figure 1. An amplifier includes the electron gun, interaction circuit, beam confinement magnets, beam collector, and radiation ports, while an oscillator does not need the input port. VEDs convert electrically stored energy into kinetic energy of an accelerated electron beam, which is then converted into an electromagnetic field energy with the aid of interaction circuits [14]. The interaction circuit determines the device’s characteristics and may, therefore, be considered the most important component of an oscillator or amplifier.

However, the reliable realization of THz interaction circuits with high performance is still challenging, that is the main reason for the least exploration of the THz frequency domain. According to the classical physics mechanisms for vacuum electron devices, the cross-sectional size of the circuit must scale with the free space wavelength. Therefore, the transverse circuit dimensions of THz vacuum electron device sources must be in sub-mm range or less [15,16].

Conventional machining and conventional MEMS microfabrication techniques are no longer the preferred solutions, because the sub-mm feature sizes are nearly too small for the former while simultaneously too large for the latter [17].

The most commonly used interaction circuits for microwave VEDs, such as coupled cavity and helix, are also not applicable for THz devices, mainly due to uncertainties in the fabrication and assembly of these small circuits, and the extremely high costs to eliminate the uncertainties. Furthermore, the intrinsic shortcomings of them, such as the poor cooling effect and low output power of helix, and the narrow bandwidth of coupled cavity, show more obvious influence at THz regime [18].

Several new interaction circuits less sensitive to fabrication uncertainties are proposed, including double corrugated waveguide (DCW) [19], double staggered grating (DSG) [20], serpentine and folded waveguide (FWG) [21], etc. The superiority of these circuits is that they are all quasi-two-dimensional structures, making their fabrication processes readily compatible with MEMS techniques, and they can be conveniently realized by both advanced micromachining and microfabrication. Moreover, these circuits can be fabricated in two halves which simply bond together, and are physically robust, cheap, and easy to manufacture [22].

**Figure 2:**
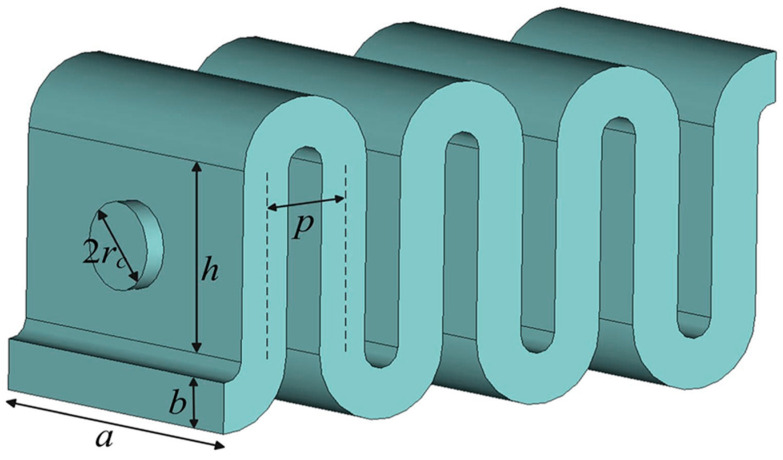
Diagrammatic illustration of a folded waveguide.

FWG is derived basically from a rectangular waveguide, by folding in their E-plane multiple times, and with a beam tunnel at the center of the straight waveguide, as shown in Figure 2. It is a periodic structure and has the dispersion characteristics similar to those of a coupled cavity. Owing to its advantages of wide bandwidth, large power capacity, and easy fabrication with microfabrication technologies, resulted from the rugged, simple coupling geometrical configuration [23,24], FWGs have attracted wide attention for developing compact THz wave radiation sources [25,26,27].

As the most representative high frequency interaction structure for terahertz VEDs, the electronic aspects of FWG have already been published by Sudhaman H. S. et al. in [28,29,30,31], while the mechanical aspects of it is also of importance for device applications. The fundamental requirements for THz interaction circuits include high-quality surfaces, high machining accuracy, high alignment accuracy with other components, high compatibility with hard vacuum, and high temperature thermal processing during manufacture.

The structure parameters affect the electrical parameters of FWG and the performance of the device. For example, the waveguide wide side (*a*) mainly influence the dispersion characteristics, the operation voltage and the working bandwidth; the waveguide narrow side (*b*) mainly influence the coupling impedance and the power loss; the waveguide period (2*p*) influence the coupling impedance; the electron beam tunnel radius (*r_c_*) influence the electron transmission, etc. In addition, there is a close relationship and mutual influence between the parameters, which should be paid more attention in design and fabrication.

There is, so far, no international standard for defining sizes and interfaces of THz waveguides. From the basic principles of the beam-wave interaction physics [32,33], an efficient beam-wave energy transfer requires the transverse dimensions of the circuit to be less than or equal to approximately 0.1*λ*_0_; meanwhile, for reliable device performance, the dimensional tolerances the circuit should be <10% error between the fabricated dimensions and the intended values [34].

The surface roughness of the waveguide wall has an important influence on the transmission loss of FWG circuit in THz regime. In the Hammerstad–Bekkadal model [35], the effective conductivity of bulk metal depends only on the surface roughness *Ra* and skin depth of the sample *δ*, and a basic requirement of low-loss transmission is *Ra* < *δ*. With the increasing roughness of metal surface, electromagnetic waves are scattered diffusely and result in a loss of power spectral density [36].

The skin depth of metal sample *δ* can be written as:

(1) $$\delta =\sqrt{2/\omega \mu \sigma }$$

where *σ* is the dc conductivity of metal sample, *ω* is the angular frequency, and *μ* is the permeability of vacuum.

According to the Equation (1), the skin depth reduces at the increase in frequency. For most of the metals adopted for microstructure fabrication, the skin depth above 100 GHz is below 200 nm, and less than 100 nm when above 500 GHz [37]. The calculated results of skin depths at different frequencies for oxygen-free copper (*σ* = 5.99 × 10^7^ S/m) are shown in Table 1. The skin depth values are the upper threshold of the surface roughness can be tolerated. With the increase in surface roughness, the ohmic losses increase and the performance degrades obviously.

A specific research example can provide better illustration of the parameter requirements for interaction circuit. For a 220 GHz FWG, achieving the tolerances of ±3–5 μm, surface roughness of 40–45 nm, and flatness, perpendicularity, squareness, and positional tolerances of ±5 μm are equally critical for the performance [38].

The strict parameter requirement for THz interaction circuits is considerably beyond the capabilities of conventional machining technologies. Therefore, novel microfabrication methods for reliable and repeatable realization of the interaction circuits become paramount [39].

The most promising microfabrication techniques for producing THz interaction structures and the applications of them in VEDs are described and reviewed in this paper, including LIGA/UV-LIGA, deep reactive ion etching (DRIE), electrical discharge machining (EDM), micro/nano computer numerical control (CNC) milling and 3D printing [40,41]. To make a better comparation, the structures are mainly focused on FWGs, and the VEDs are mainly focused on travelling wave tubes (TWTs) [42,43]. Additionally, some other applicable techniques, such as electrochemical milling, focused ion beam milling, and laser etching/drilling, are not included due to the lack of literature.

## 2. LIGA/UV-LIGA

### 2.1. LIGA

**Figure 3:**
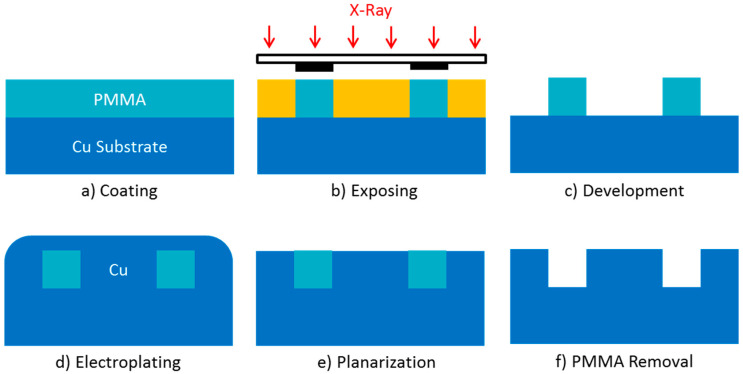
Process flow diagram of LIGA.

LIGA is a German acronym of “Lithographic, Galvonoformung and Abformung”, standing for lithography, electroforming, and molding. LIGA technology is a micro manufacturing process developed in the early 1980s; it is one of the main technologies that enables on-demand manufacturing of high aspect ratio structures with a lateral accuracy of less than one micrometer [44]. A typical LIGA process is as follows, as illustrated in Figure 3.

X-ray sensitive polymer photoresist PMMA is coated onto a conductive copper substrate, and then exposed to parallel high-energy X-ray beams emitted by a synchrotron radiation source through a mask, which is partially covered with a strong X-ray absorbing material, gold. Remove exposed PMMA through chemical methods to form a three-dimensional structure, and then fill it with electrodeposited copper. After the photoresist is chemically peeled off, a copper mold insert is produced [45]. The mold inserts can be the required structures or be further used to produce polymer or ceramic components through injection molding.

A unique characteristic of LIGA is a large penetration depth of the X-ray, making it possible to fabricate high aspect ratio structures with very small dimensions, which is unmatched by either micromachining or MEMS process. The typical fabrication tolerance of it is about 1 μm, the high aspect ratio is up to maximum 100:1, and the size covers micron and submicron ranges in horizontal dimensions, and millimeter and centimeter ranges in vertical dimensions [22,46,47]. These dimensions are ideal for fabrication of interaction circuits with frequencies from 30 GHz to 1 THz [48].

**Figure 4:**
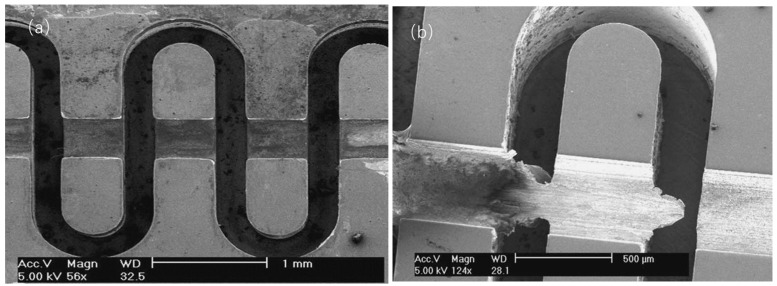
Scanning electron microscope images of (**a**) two-step LIGA-fabricated FWG and (**b**) post-LIGA machined FWG [49].

By using a two-step LIGA process, Y. M. Shin et al. fabricated a FWG structure for W-band TWT [49]. The first LIGA process layer is only one part of waveguide and the second layer is the other part of the waveguide with beam tunnel. The two-step LIGA-fabricated circuit has accurate dimensions and clear edges, as seen in Figure 4a. Meanwhile, the conventional post-LIGA machined structure has clogged waveguide paths around the beam tunnel due to the burs planed off from a partially torn LIGA-metal block, as seen in Figure 4b. The FWG circuits have a measured tolerance of below 2 μm and a surface roughness of 20–70 nm, implying that the two-step LIGA microfabrication has a good application potential.

**Figure 5:**
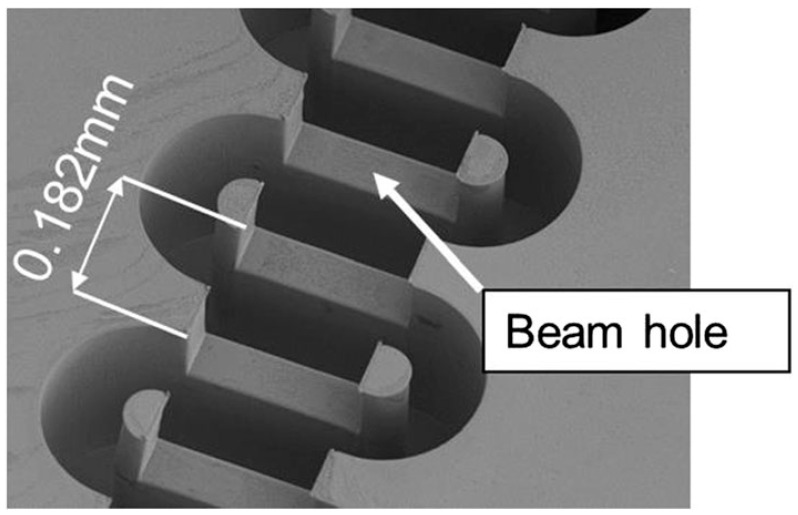
Trial 300 GHz FWG fabricated with X-ray LIGA [50].

K. Tsutaki et al. manufactured FWGs for a 300 GHz TWT by the two-step LIGA process, in order to achieve output power greater than 1 W and gain larger than 20 dB. The dimensional accuracy of the structure met the design requirement well, as seen in Figure 5 [50].

**Figure 6:**
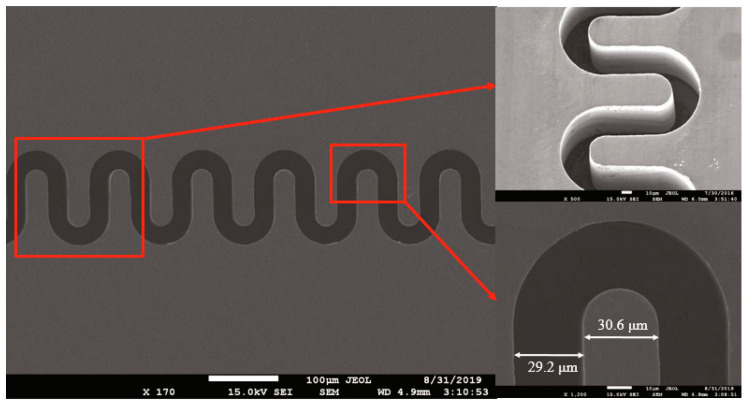
SEM photo of 850 GHz FWG circuit; scale bar, 100 μm; scale bar in insert, 10 μm [51].

K. H. Jang et al. designed and fabricated W-band, G-band, and 850 GHz FWGs for TWT amplifiers by using LIGA process. The test results show that the transmission loss and return loss of the W-band circuit are 0.42 dB/cm and −10 dB, respectively; and the transmission loss of the G-band circuit is 1.57 dB/cm. Figure 6 shows the fabricated 850 GHz metal FWG. The tolerance of horizontal critical dimensions is about ±1 μm and the vertical angle is close to 90°, indicating an excellent resolution compared to those of DRIE and UV-LIGA. The arithmetic average surface roughness is assured to be 40 nm, which is comparable or superior to those surfaces fabricated by micro/nano-CNC milling and wire-EDM [51].

LIGA is a promising and robust candidate for precise microfabrication of THz interaction circuits, with high aspect ratios and good vertical angles. However, x-ray illumination requires a large synchrotron facility, and the beamline is not readily accessible at all times. Usually, it takes several weeks to complete a whole LIGA process of an interaction circuit. High costs and long development cycles are major application hurdles of LIGA, thereby reducing its attractiveness for interaction circuit fabrication [52,53].

### 2.2. UV-LIGA and EDM

A considerably less expensive and less time-consuming option to LIGA is UV-LIGA, where ultraviolet (UV) radiation rather than an X-ray is used for the exposure of thick film photoresists. The UV-LIGA process has degraded aerial images when compared with LIGA technique, mainly because of its much longer wavelength. X-ray has a typical equivalent wavelength in 0.01–10 nm, it can directly penetrate PMMA thickness up to one millimeter easily, and realize a tolerance of 1 μm or better. While the UV for lithography is usually with a wavelength of 365 nm, and the UV light is uncollimated in thick photoresist layer due to the diffraction or absorption, which makes UV-LIGA a slightly inferior tolerance of 3–5 μm. However, the tolerance is still considerably superior to those attainable in traditional machining techniques [54].

SU8 is a commonly used negative-tone photoresist in UV-LIGA process. It becomes attractive for many applications in high-aspect-ratio microfabrication because of its high sensitivity to UV radiation and its good resolution, which make it produce high resolution features with superior sidewall fidelity [55]. SU8 photoresist has a much faster exposure time with a UV source, and a typical SU8 UV-LIGA process only requires several days, which is attractive for the fabrication of THz interaction circuits [56].

O. V. Makarova et al. proposed to prepare low frequency THz FWG by the combination of UV-LIGA and EDM. The FWG structures are fabricated by UV-LIGA to realize high dimensional accuracy and smooth vertical side walls, while the electron beam tunnels are realized by EDM to further simplify the fabrication process [57].

H. Li and J Feng et al. fabricated W-band FWGs by using UV-LIGA and EDM process. The height and width dimensional accuracies of the FWGs are ±2 μm and ±10 μm, respectively. The sidewall verticality of FWGs is about 90.3°, and the sidewall roughness is ranged from 34 nm to 200 nm [58,59]. Based on the FWGs, a Y-band TWT 2nd harmonic amplifier was developed. The amplifier shows over 100 mW output power with a bandwidth of 11.4 GHz at duty cycle of 1% [60]. Also based on this kind of FWGs, J Cai [61] and Y. Du [62] et al. developed W-band pulsed TWTs. The prototype TWTs show a gain of over 30 dB and saturated output power of over 100 W.

**Figure 7:**
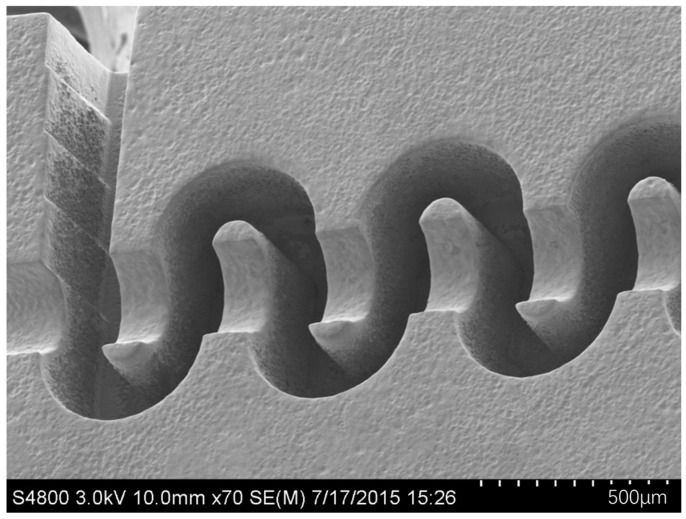
220 GHz FWG fabricated by UV-LIGA with electron channel fabricated by EDM.

H. Li et al. then extended the UV-LIGA and EDM technology to 220 GHz FWGs, as seen in Figure 7. The height accuracy of the fabricated FWG is 10 µm, the width accuracy is 6 µm, and the surface roughness of the sidewall is 56 nm [63]. P. Pan et al. employed the 220 GHz FWGs into a compact TWT, realizing 10 W output power and 10 GHz bandwidth [64].

### 2.3. Multi-Step UV-LIGA

According to the two-step LIGA process of Y M Shin [49], a similar multi-step UV-LIGA process was developed. The process is expected to produce higher quality electron beam tunnels with better alignment, when compared with the combining process of UV-LIGA and EDM.

**Figure 8:**
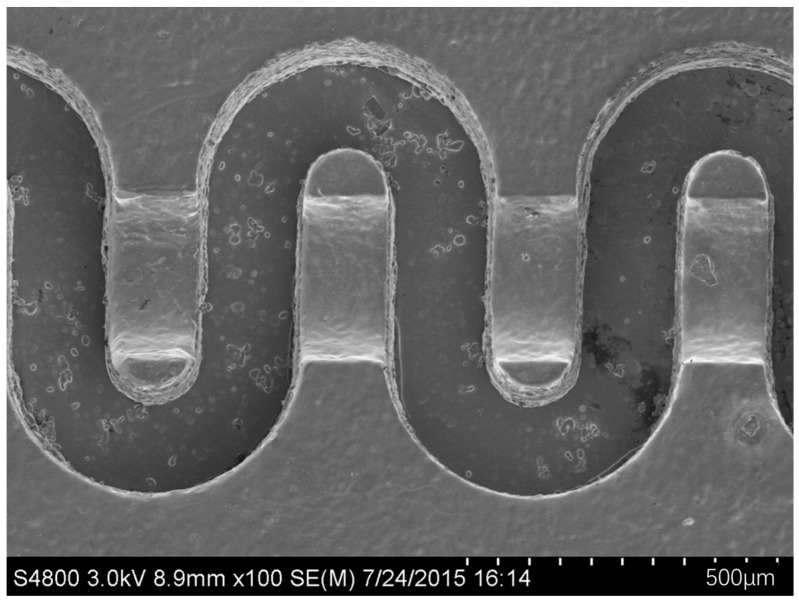
W-band FWG fabricated by two-step UV-LIGA process.

H. Li et al. fabricated W-band FWGs by using the process. As seen in Figure 8, the line width tolerance of the mold is ±3 µm, the surface roughness is 100 nm, the side-wall profile is 90.5°, and the alignment error between the two layers is ±0.1 µm [65]. A pulsed TWTs based on the FWGs is developed. It has an output power over 100 W, bandwidth of 6.7 GHz, saturated gain over 33 dB at 20% duty cycle [66].

F. Xie et al. adopted the technology to a 140 GHz TWT. The tolerance of the FWG is less than 20 μm, the roughness of the inner sidewall is about 400–500 nm. And the continuous output power of the TWT is over 25 W, with a gain close to 23 dB [67].

P. Pan et al. employed the multi-step UV-LIGA FWGs in a 220 GHz TWT. The tolerance of the FWGs is 10 μm, and the output power of the TWT is 10 W with a bandwidth of 10 GHz [68].

Y. Xie et al. and L. Yao et al. employed the process to make 340 GHz FWGs, respectively. Although FWGs with process deviation less than 1 μm and good surface roughness were obtained, there were no corresponding TWTs reported [69].

Also using the multi-step process, O. V. Makarova1 et al. realized reproducible fabrication of all-copper FWGs for 0.67 THz amplifier. The sidewalls roughness of the SU-8 mold is below 50 nm, and the horizontal and vertical accuracy along the copper waveguide length are within 1 µm [70].

S. Qin et al. tried to make 850 GHz FWGs. The waveguide part has been realized and the electron tunnel is ongoing by the second step UV-LIGA. With an optimal process, the tolerance of the fabricated waveguide is less than 1 µm, the surface roughness of the inner sidewalls is less 20 nm, and the profile is near to 90° [71,72].

### 2.4. Embedded Monofilament UV-LIGA

The multi-step UV-LIGA process always produces square beam tunnels. However, a round beam tunnel is preferred in the THz range, because the oversized square beam tunnels result in severe gain loss and increase unwanted coupling to adjacent slots.

**Figure 9:**
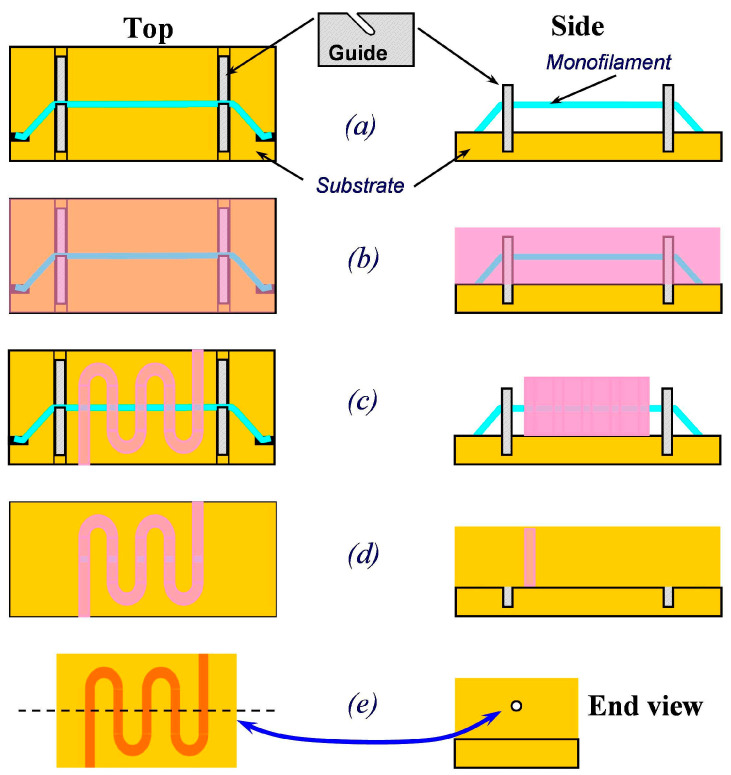
Embedded polymer monofilament concept. (**a**) The monofilament is set, (**b**) photoresist is applied, (**c**) UV-exposure and developing, (**d**) electroforming metal, (**e**) removal of the monofilament and photoresist [73].

With an embedded polymer monofilament UV-LIGA patent process proposed by C D Joye [73], all-copper FWGs can be fabricated with arbitrarily small beam tunnels and arbitrary length along with interaction circuits. As shown in Figure 9, the process relies on the embedding of a UV-transparent polymer monofilament into the photoresist before exposure, wherein the filament preserves the shape of the beam tunnel, mitigating the need for sinker EDM, drilling, the second step UV-LIGA, or other limited methods. 

A major advantage of the process is that both the beam tunnel and interaction circuit are simultaneously formed in a single exposure step. In addition, a variety of beam tunnel shapes can be easily envisioned, including multiple round beams, ellipses, sheets, squares, etc.

A. M. Cook et al. first employed the embedded monofilament UV-LIGA process in the fabrication of FWGs for W-band TWTs [74,75]. The depth of the 95 GHz waveguide is 1700 μm and the width is 252 μm, with an aspect ratio of ~7:1. By using a multiple-layer technique, the aspect ratio of each layer is well managed, and a small side-wall angle of ~0.7° is obtained.

C. D. Joye et al. used the process in G-band TWTs. The thickness of SU-8 layer is over 850 μm with aspect ratios up to 9:1. The TWT has output power over 60 W at 214.5 GHz, and the small signal instantaneous bandwidth is about 15 GHz [76,77].

Q. Liu et al. used the process in 310 GHz TWTs. The dimensional accuracy of the interaction circuit is less than 3 μm and the surface roughness is less than 30 nm, both of which meet the requirements of the TWTs. But there is no further report on assembly and welding technology [78].

The limitations of the embedded polymer monofilament UV-LIGA process are mainly the available of thin monofilament, the fabrication precision of filament guides, and the alignment errors of the filament.

### 2.5. KMPR UV-LIGA

SU8 realizes sufficient crosslink in exposure process, which can repel swelling in organic developers. However, the highly cross-linked SU8 has robust properties, and the removal of SU8 in UV-LIGA structures is a traditional, albeit troublesome, problem [79].

Various methods are attempted to solve the problem, including chemical erosion, plasma etching, molten salts, and so on [80,81,82,83,84]. Nevertheless, there is not an ideal method that can completely remove the SU8 without affecting the electroplating metal structures.

To overcome the disadvantages of SU-8, some alternative photoresists are tried in UV-LIGA process, and KMPR is the one similar to SU-8 but is much easier to remove [85,86]. One comparison between KMPR and SU8 shows that, in a 120 μm deep electroplated copper structure, the cross-linked KMPR mold is completely dissolved and removed, whereas the SU8 mostly remains and sticks to the copper structure [87].

A 400 μm thick KMPR mold is demonstrated by single spin coat, with fairly vertical sidewalls of less than 1° angle deviation and reasonably small undercuts. The electrode-posited copper structure has surface roughness of ∼50–100 nm and dimensional accuracy of ±3 μm, and the photoresist mold can be chemically stripped off from the metal structure without noticeable damage [88].

H. Li et al. fabricated a 340 GHz FWG by using the KMPR process. The dimensional accuracies of the height (500 μm) and width (100 μm) of the copper FWGs are less than 6 μm and 2 μm, respectively; and the sidewall surface roughness is about 44 nm, with a profile of about 90° [89].

L. R. Billa et al. adopted the process to realize the interaction circuit of a 400 GHz TWT amplifier. Measurements of the samples show that the dimensional accuracy is within 2.5 μm, and the sidewall surface roughness is less than 75 nm [90].

KMPR is well known for its excellent chemical solubility after electroforming. However, it is more sensitive to the UV exposure dose, which will result in inconsistent mold quality in high aspect ratio circuit structures: delamination of the photoresist from the substrate, poor wall verticality, and poor dimensional accuracy. KMPR is also much softer than SU-8, which can potentially lead to deformation during the lapping and polishing processes.

In general, LIGA process can realize excellent fabrication accuracy and surface roughness, while the high costs and long development cycle restrict its application mainly in scientific research. UV-LIGA is an effective alternative for LIGA, it has been used in W-band and G-band TWTs, and has promising application potential in higher frequencies.

## 3. DRIE

**Figure 10:**
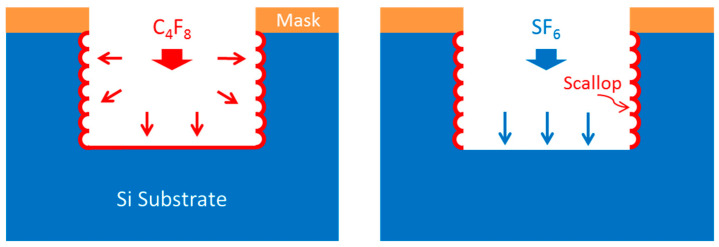
Schematic of Bosch method: Sidewall passivation using C_4_F_8_ and silicon isotropic etching using SF_6_.

Deep reactive ion etching (DRIE) is also a suitable process for interaction circuit fabrication. It is an anisotropic reactive ion process for microfabricating high aspect ratio structures in silicon. The etching mechanism of DRIE can be illustrated by a patented Bosch method. As seen in Figure 10, it relies on many repeated cycles of etching followed by passivating sidewalls via polymerization [91,92,93,94].

In a passivating cycle, only C_4_F_8_ is used, and a Teflon-like film is deposited on the bottom and sidewalls. During the subsequent etching cycle, the passivating film is preferentially removed from the bottom due to SF_6_ ion bombardment and reaction, while the etching of the sidewalls is prevented. The alternating of etching and passivating cycles forms scallops on the sidewalls of etched features.

The key specifications of DRIE process are high etching rate, straight trench sidewall profile, smooth surface patterns, and low etch loading effects, and they can be controlled by an optimal etching process [95].

K. S. Chen et al. proved the anisotropy, etching uniformity, fillet radii, and surface roughness are achievable by the control of chamber pressure, applied coil and electrode power, and reactant gases flow rate [96].

Miller K. et al. indicated the effects of etching cycle time, passivating cycle time and platen power, on the structure profile angles and scallop depths. By using an optimized DRIE process, nearly 90° and smooth surface (invisible scalloping) can be created [97].

C. Jung-Kubiak et al. developed a DRIE process using multiple SiO_2_ masks to enable multi-depth features with ±2% tolerance. By adding a thermal oxidation process after the completion of silicon structures, the sidewall roughness is controlled to be several decade nanometers [98].

**Figure 11:**
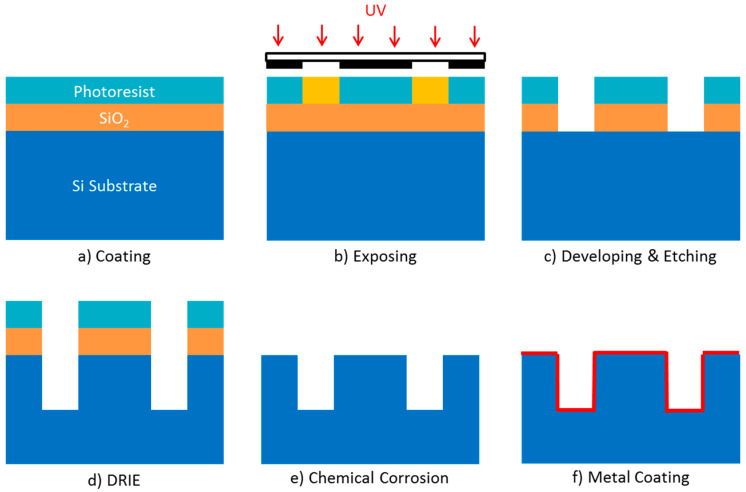
Process flow diagram of DRIE for interaction circuit fabrication.

DRIE-based micromachining of bulk silicon wafers is a well-established fabrication technique capable of etching high aspect ratio features. A typical DRIE process for interaction circuit is as follows, illustrated in Figure 11.

A silicon substrate with a SiO_2_ layer is coated by photoresist and subsequently exposed to parallel UV beams through a mask. The exposed photoresist is developed and the beneath SiO_2_ is etched down to silicon substrate. Through the etched SiO_2_, the silicon is etched to a desired depth by DRIE process. The residual SiO_2_ and photoresist are removed and the final structure is obtained. To be used as an interaction circuit, a blanket film of low-resistivity metal needs to be coated on the inner surface of the silicon structure.

A silicon micromachined W-band FWG, by using DRIE process, was first realized by Y. Li et al. [99]. The measured insertion loss for the FWG was between 0.41 dB/cm–0.69 dB/cm over 90 GHz–110 GHz. The result shows the waveguides have competitive performance compared to a commercial standard waveguide.

H. Li et al. developed a W-band FWG by the DRIE process. The serpentine trench depth is approximately 946 μm and the profile is 91°; the beam tunnel depth is approximately 225 μm and the profile is 90°. A 1.5 μm copper layer is evaporated inside the serpentine trench for better wave transmission [100,101].

C. W. Baik et al. proposed a two-level μ-DRIE process on multi-bonded silicon wafers to fabricate FWGs with beam tunnel. A W-band interaction circuit with a high aspect ratio of 8:1 is obtained, the surface roughness of the sidewall is as low as 13.8 nm after a surface smoothing process, which usually includes thermal oxidation and chemical etching. The return loss measurement indicates not only the dimensions but also the surface roughness can meet the device requirements [102,103].

Y. M. Shin et al. fabricated a 220 GHz DSG by DRIE process. The dimensional tolerance of the pattern on the top of the 770 μm tall fin is less than 1 μm, the sidewall slope is of >89.6°, and the surface roughness is about 30 nm after a thermal oxidation and etching process [53].

A 220 GHz VED power amplifier is designed and demonstrated by M. A. Basten and K. E. Kreischer et al. A two-level DRIE process is used to etch both the trench and the beam tunnel of the FWGs into a silicon-on-insulator (SOI) substrate. The use of SOI wafers can precisely control the FWG depth and eliminate the saddle-shaped bottom surfaces. The amplifier demonstrates 56 W at 214 GHz and a 5.0 GHz bandwidth in short pulse tests [104,105].

K. E. Kreischer et al. employed the two-step process in 650 GHz FWGs. The waveguide aspect ratio exceeds 8:1, with height accuracy of 5% and height uniformity better than 1%. The sidewall roughness is 50 nm, and the alignment accuracy of the two FWG halves is 2 μm. Based on the circuit, a compact 650 GHz vacuum electronic source was realized, which has an output power of 50 mW, an interaction efficiency of 0.45%, and a duty cycle of up to 3% [106].

Based on the well-built two-step DRIE process, 670 GHz, 850 GHz and 1.03 THz TWT amplifiers were developed in succession by J. C. Tucek and M. A. Basten et al. [107,108,109,110,111].

The 670 GHz FWG has a high aspect ratio of 8: 1, sidewall surface roughness of 50 nm, and alignment accuracy of 0.5 μm. A power module based on the 670 GHz TWT can produce > 100 mW output power in the range of 640–685 GHz [107,108].

A dual-SOI wafer is adopted to act as the depth and beam tunnel etch-stop of an 850 GHz FWG. It is expected to improve the dimensional control of the beam tunnel through the length of the circuit. The corresponding TWT produces output power > 50 mW in 835–842 GHz and 39.4 mW at 850 GHz. The small signal gain is 26 dB and the instantaneous bandwidth is 11 GHz [109,110].

The 1.03 THz FWG circuit has a critical dimension less than 10 μm, which is only achievable through the most advanced DRIE technique at present. The 1.03 THz TWT produces 29 mW at 1.03 THz, with a gain of 20 dB and instantaneous saturation bandwidth of 5 GHz [111].

R. Yang et al. tried to use DRIE process in 1 THz interaction circuit. The measurement shows that the height tolerance of the waveguide and the beam channel are all around 0.8 μm, the width tolerance of the waveguide is 0.3 μm, and the surface roughness ranges from 27 nm to 41 nm. The interaction circuit can meet the design requirement, but there is no corresponding device reported [112].

**Figure 12:**
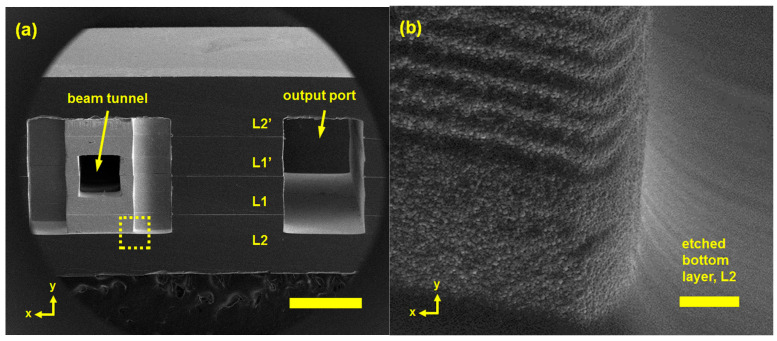
(**a**) Cross-sectional SEM image of a multi-level interaction circuit; scale bar, 1 mm; (**b**) SEM image of etched bottom layer; scale bar, 5 μm [115].

To achieve better high-respect ratio silicon structure, C. W. Baik et al. proposed a DRIE-based multi-level fabrication process. By splitting a high-aspect ratio interaction circuits into multilevel, the features including the beam tunnel, the waveguides, and the output port can be precisely prepared separately. The low-aspect ratio configuration can alleviate the loading effect of deep etching on silicon wafers, so that rather flat-etched bottom and smooth sidewall profiles with nanoscale accuracy can be achieved, as seen in Figure 12. In addition, it permits fairly sophisticated 3D structures, by using monolithic integration [113,114,115].

In summary, DRIE is one of the most developed MEMS technologies. Although the poor thermal conductivity and electric conductivity of silicon restrict the output power of devices, the DRIE fabricated silicon interaction circuit is the only successful application in actual VEDs above 1 THz up to now.

## 4. Micro/Nano CNC Milling

Micro mills are unique and important micro tools for miniaturized devices. The well-designed micro milling cutters can achieve high machined surface integrity and dimensional accuracy, the multiple axis controlling can manufacture complex machining contours and challenging structures, without special requirements on workpiece material properties [116,117,118,119].

The computer numerical controlled (CNC) milling is an attractive microfabrication technique, it has advantages of shorter fabrication time, cheaper cost, and competitive precision [120,121,122]. However, due to the limitation of tool size, the conventional CNC milling, can be used only for features larger than 500 μm.

With the advancement of milling tools and manufacturing programs, the State-of-the-Art micro/nano CNC milling machines have been reported for manufacturing micro-scale components with high precision. They are theoretically suitable for the processing of micro-groove structures, which are usually the main parts in THz interaction circuits [123,124,125,126].

When working on the THz interaction circuits by CNC methods, there are some challenges to be considered, such as the small cutting tool, the micron scale tolerance, the sharp cornered features and the very low surface roughness.

Based on the specialized precision micro tooling and the specialized nano manufacturing program, a NN1000 DCG HSC nano-CNC machine developed by the Digital Technology Laboratory, DMG Mori, Davis, CA, USA, is expected to realize submicron features with high repeatability (±0.5 µm) and excellent surface roughness (50 nm Ra) [127].

D. Gamzina and L. Himes et al. [128] demonstrated some interaction circuits fabricated by the machine, as summarized in Table 2. The circuits include a coupling cavity (CC) for 94 GHz sheet beam klystron (SBK), double staggered gratings (DSG) for sheet beam (SB) TWTs and sheet beam backward wave oscillators (BWO) with different frequency, and a double corrugated waveguide (DCW) for 346 GHz pencil beam BWOs, and a folded waveguide (FWG) for 270 GHz pencil beam TWT.

All the fabricated structures can assure the dimensions and roughness to the requirements. For the same structures, the features manufactured by using smaller tools had worse surface finish than those by large tools. This can be attributed to the significant increase in tool deflection due to the reduced tool stiffness.

With a micro/nano CNC milling fabricated FWG, W Q Lei et al. developed a D-band continuous-wave (CW) TWT. The output power is 7.3 W at the center frequency of 140.3 GHz, with a gain of 25.3 dB and a 3 dB bandwidth of 3 GHz [129,130].

W. Liu et al. fabricated a two-section FWG for 220 GHz TWT by this process. The processing errors of the FWG are within 2–5 μm. The cold test results agree well with theoretical predications, but the roughness is required to be improved [131].

H. S. Sudhamani and R. K. Bhardwaj et al. carried out 220 GHz FWGs with circular and rectangular beam holes. The fabrication tolerances of the FWGs are within ±3–5 μm, the surface roughness is of 45 nm, the aligning tolerance is of ±5 μm, and the perpendicularity and parallelism are all within 5 μm, which can meet the design requirements [31].

Based on a micro/nano CNC milling FWG, Q. Zhou et al. developed a 0.22 THz broadband TWT amplifier. Under pulse width of 50μs and pulse repetition of 100 Hz, the peak power of the TWT is 350 mW, the gain is 16 dB, and the instantaneous 3 dB bandwidth is 8.8 GHz [132].

P. Pan and X. Bian et al. proposed a FWG with modified circular bends and fabricated it by micro/nano CNC milling. On this basis, CW and pulsed G-band TWTs were realized. The maximum output power of the CW TWT is over 20 W, the transmission ratio is over 95%, and the efficiency is over 5%; while the output power of the pulsed TWT is over 50 W, the electronic efficiency is higher than 3.5% and the gain is higher than 35 dB [133,134].

M. A. Basten et al. developed a vacuum electronic high-power amplifier at 233 GHz. The amplifier demonstrates an output power greater than 50 W and a saturated gain approximately 24 dBm over a 2.4 GHz instantaneous bandwidth. The typically beam transmission is about 95–98%, and the operational duty cycle is up to 50% with liquid cooling [135].

P. Hu et al. fabricated interaction circuits for TWTs operating above 300 GHz. For a 320 GHz FWG, the dimension tolerance is ±5 µm, the surface roughness is 300 nm. The corresponding TWT demonstrates maximum output power over 130 mW and small-signal gain of 19.6 dB, with a duty cycle of 10% [136]. For a 340 GHz FWG, the dimension tolerance is ±3 µm, the surface roughness is 300 nm. The TWT achieves output power over 3.1 W and gain of 26.2 dB [137].

**Figure 13:**
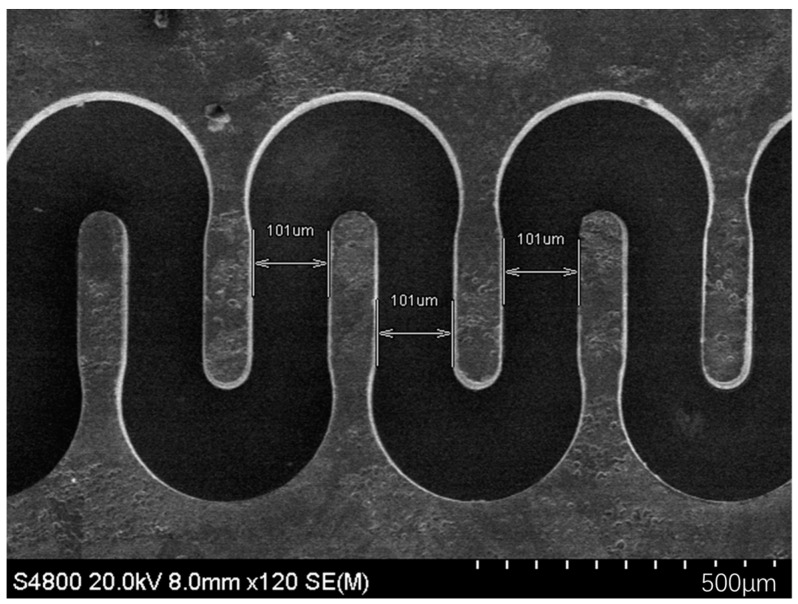
340 GHz FWG with modified circular bends fabricated by micro/nano CNC milling.

L. Zhang et al. developed a 0.34 THz TWT based on a FWG with modified circular bends. The FWG is seen in Figure 13, it has tolerances of less than ±3 μm and inside roughness less than 100 nm. The TWT achieves output power over 6 W at 0.334 THz, with gain over 39 dB, and 3 W output power in 10 GHz bandwidth [138].

Based on a number of prototypes of THz VEDs, C Paoloni and J Feng claimed that the micro/nano CNC milling is effective up to 0.35 THz and above [20,139].

Modified sine waveguides were proposed to ease the fabricating difficulty of FWGs [140]. W. Choi et al. fabricated a sine waveguide for 300 GHz TWT, by using a nano CNC machine with a 0.12 mm diameter tool tip. The maximum fabrication error is estimated to be ±6 μm, and the simulation indicates a maximum output power of 10 W with an ideal injected electron beam [141].

S. Fang and R. Yang et al. fabricated 0.38 THz and 1 THz sine waveguides [142,143]. For the fabrication of 1 THz waveguide, the diameter of the endmill cutter is 48 μm and the length is 76 μm. The surface roughness of the waveguide is around 58–74 nm, which is roughly equivalent to the skin depth. The beam-wave interaction simulation results based on the cold test results show that the TWT has 300 mW output power, and the 3 dB bandwidth is around 3 GHz.

Compared to UV-LIGA or DRIE, micro/nano CNC milling is capable of machining complex structures at the micro- and meso-scales. However, tool wear and size effect limit its further application [144].

Due to the tool wear, more than one micro tool is needed to produce one S-shaped micro-groove with 100 cycles and above. Tool change causes additional tool alignment error, and the miniature difference in tool sizes inevitably reduces both the machining accuracy and surface quality. Combined machining processes were carried out to solve the unavoidable problems, including laser-assisted micro-machining [145,146,147], laser-induced oxidation assisted micro milling [148], nanosecond laser hybridizing with micro-milling (NLMM), etc.

In a NLMM process, the nanosecond laser achieves the maximum workpiece removal rate, while the subsequent micro-milling provides the desired machining quality. By using the NLMM method, X. Hao et al. fabricated a desired S-shaped groove, with an aspect ratio of 2.5, a width of 0.2 mm, and a cycle number of 30 on oxygen-free copper [149].

Based on a novel modified staggered double-vane interaction circuit fabricated by this NLMM process, P. Pan and L. Zhang et al. developed a 0.34 THz CW TWT as well as a microwave power module (MPM). The MPM provides an output power over 1 W and a gain over 18 dB from 0.33 THz to 0.355 THz [150,151]. Obviously, the NLMM process can be easily applied in W-band and G-band and has potential for frequency ranges above 340 GHz.

With the improved machines, tooling, and computer software, micro/nano-CNC milling becomes an attractive microfabrication technique for THz interaction circuits. It has been used in VEDs with frequencies up to 340 GHz and may be more competitive in higher frequency ranges with the further reduction of milling tool size.

## 5. Three-Dimensional Printing

Three-dimensional printing technology has become popular, not only due to the lower cost and weight but more significantly the ability to create complex geometries in a simple fashion. In addition, it provides a very large array of materials from plastic filaments to ceramic resins and to metallic powders, enabling a vast array of applications in microwave systems.

Direct 3D printing of fully dense metal structures has been demonstrated for a wide range of materials. It builds the part layer by layer, fusing metal powder into a solid by melting it locally using a focused high-energy beam [152].

The Direct 3D metal printing technology was investigated for a 35 GHz TWT amplifier by J. P. Anderson. The results show that the printed circuits have good electrical performance but with large surface roughness [153]. Without an infiltrating material to fill in the porous metal, metal printing is unable to produce pure copper parts, and the resolution of printed features is relatively coarse.

T. Horn et al. verified the above results. The WR-10 waveguides fabricated by 3D direct printing have an average surface roughness of 28–44 μm, far greater than the value of skin depth [154]. It shows that, for the implementation of 3D direct metal printing techniques in VEDs, post-processing such as magnetic slurry polishing and abrasive micro-fluidics magnetic slurry polishing are essential [155]. By using a magnetic slurry polishing with appropriate compound fluid slurries and optimum process parameters, nano-precision surface finish of oxygen-free copper can be realized [156].

Three-dimensional polymer-based printing is also a popular additive manufacturing technology, which can realize light, smooth structure with higher resolution when compared with 3D metal printing. However, the polymer mold cannot be used as vacuum electronic components directly, unless a robust metal layer is coated on its surface.

J. Shen et al. proposed to make a complex magic tee waveguide by using 3D polymer-based printing and post metallization process. The printed W-band magic tee has a comparable performance to a CNC-machined magic tee, but weighs 77.5% less than the metallic one [157].

A. M. Cook et al. fabricated W-band and D-band FWG circuits for TWTs by using 3D polymer-based printing, respectively. The circuits were fabricated by using a commercial digital light processing 3D printer, and covered with conducting layers by Au sputtering and Cu electroplating subsequently. The measured variation and error in structure parameters amount to ≤5–10 µm in absolute value, which are comparable to those typical of precision CNC machining [158].

However, the 3D polymer printed structure is not suitable for VEDs because of the material reason. In addition, the metallization of printed polymer mold is still challenging, especially when the frequency extends to the terahertz-wave, where the feature size is very small.

To eliminate the effect of polymer material, the 3D printing technology is being explored for use in a UV-LIGA-style electroforming process. In this method, the vacuum geometry of the circuit is created in a polymer material. The polymer mold is then adhered to a metal substrate, electroformed, and ultimately removed to leave a pure metal circuit.

A. M. Cook et al. used the process to fabricate W-band FWGs, and a repeatability of ~0.5% variation was realized in critical circuit dimensions such as the period and the depth of the FWGs. Although the surface of the mold looks smoothy, the electron micrographs reveal the layered nature of the printing process, indicating the 40 µm-layer-thickness is coarse compared to the beam tunnel size [75,159].

The situation with lower layer thickness is much better. A W-band FWG polymer inverse mold obtained by a 15 µm-layer-thickness process, with the consequent Cu electroformed structure were also fabricated by A. M. Cook et al. Much better accuracy and precision of critical dimensions are realized, with statistical variation of approximately 0.5% from the design values. The results can meet the requirement of TWT but have less precision than those achieved by current state-of-the-art techniques, such as micro/nano CNC machining and UV-LIGA [160].

Although 3D printing is not satisfactory in THz FWGs preparation, it has obvious advantages in achieving more complex structures. By using 3D photopolymer printing with subsequent metallization, M. D. Proyavin et al. fabricated a profiled helical waveguide for a microwave undulator, and a periodic slow-wave system for a ribbon beam BWO, respectively. The cold measurements of both structures show good agreement with the simulation results [161].

With a similar process (liquid crystal photopolymerization and magnetron sputtering), A. V. Starodubov et al. realized single grating slow-wave structures for W-band vacuum-tube devices. The morphological and profilometric analyses show that, a 3 µm metallization layer by magnetron sputtering can decrease the surface roughness of the polymer mold up to 50%, and it is sufficiently to meet the requirements for suitable reflection and transmission losses in W-band [162].

One of the most representative interaction circuits realized by 3D printing is helix, which demonstrates its fabrication ability for complex structure well.

**Figure 14:**
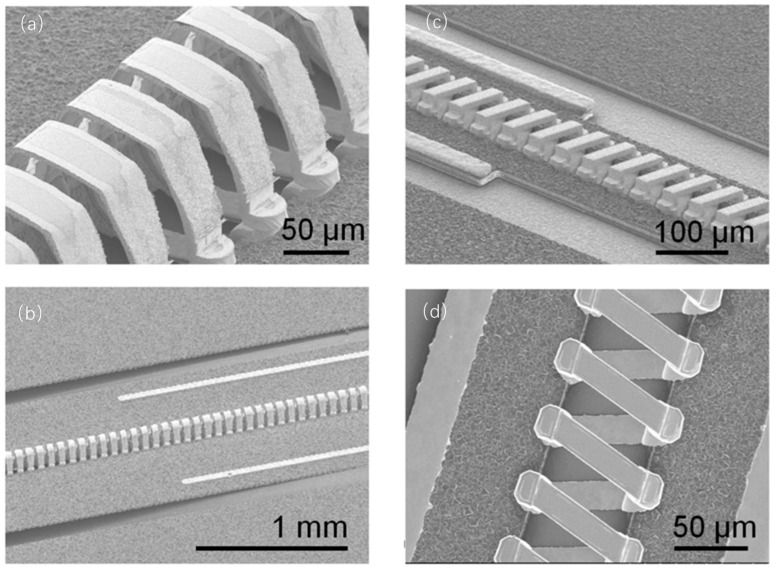
SEM images of helical slow-wave circuits for (**a**,**b**) the 95 GHz TWT and (**c**,**d**) the 650 GHz BWO [164].

With the increase in operating frequencies, the dimensions of the helix for TWTs need to be reduced, and the helix by traditional machining is hard to overcome the frequencies of 50–60 GHz [163]. M. R. Lueck had microfabricated helix slow-wave circuits for 650 GHz BWO and 95 GHz TWT, as seen in Figure 14. However, the fabrication flow is rather complicated and difficult. It consists of a lot of steps, including chemical vapor depositing, magnetron sputtering, lithography and etching, reactive ion etching, vacuum evaporating, electroplating, ion milling, corrosion releasing and bond assembling [164].

Relatively, 3D printing for helix fabrication is simple and efficient. G. Ulisse et al. realized the helix for a TWT operating in 60–80 GHz. In the process, only the horizontal structures contacted with substrate are fabricated by photolithography, all the other parts are formed by 3D printing. The measurements of the structure reveal an RF copper conductivity of about 2 × 10^7^ S/m, which is comparable to bulk copper conductivity in the millimeter-wave range. Simulations show that a TWT based on the helix has a gain of about 30 dB at 66 GHz and a 1 dB bandwidth of about 5 GHz [165].

Even if the results still need to be improved, 3D printing is demonstrated as a promising method for micro-sized electromagnetic components, due to its affordability and significant time savings. Furthermore, it has specific advantages in complex structure fabrications.

## 6. Planar Interaction Circuits and Fabrications

Compared to helix or FWG, planar interaction circuits are more attractive due to the simplicity of their structure, compact dimensions, low operating voltage, and capability to accommodate a high-aspect-ratio sheet electron beam. VEDs with sheet electron beams are research hotspots in recent years because they are capable of producing higher output power.

**Figure 15:**
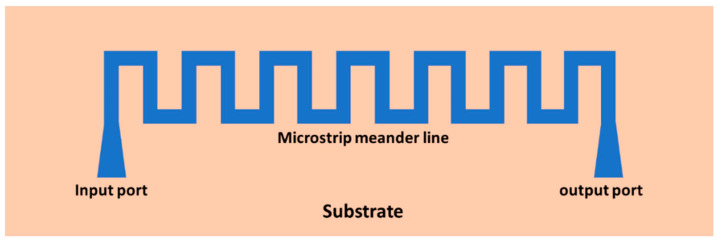
Schematic of planar microstrip meander line slow-wave structure.

The most commonly used planar interaction circuit is microstrip meander line (MML), which is the patterned metal film on a dielectric substrate or on dielectric supports, as illustrated in Figure 15.

MML can be fabricated by using advanced microfabrication techniques, such as LIGA, UV-LIGA, DRIE, and precision laser micromachining technology [166]. Meanwhile, due to the planar thin film structure, it also can be realized by simple planar microfabrication method, which only includes photolithography, magnetron sputtering, and lift-off process [167].

MML structures represent an important research direction of interaction circuits for high frequency VEDs. Up to now, MMLs with different shapes for different frequencies have been designed, fabricated and cold tested, including Ka-band [168,169], V-band [170,171,172], W-band [173,174] and D-band [175].

However, the research of actual sheet electron beam devices based on MMLs is still in simulation stage. Analysis suggests that the difficulty mainly lies in devices rather than in interaction circuits. For example, the formation and effective focusing of sheet electron beams with high current density is still an obvious obstacle but difficult to overcome.

## 7. Conclusions

A brief comparation of the reviewed microfabrication technologies for THz interaction circuits is shown Table 3.

LIGA is an excellent candidate for precise microfabrication of THz interaction circuits with high aspect ratios, smooth inside surface and good vertical angles. Efforts have been made to realize interaction circuits of 95 GHz, 300 GHz, 600 GHz, 850 GHz, and good results are obtained. But there are seldom activities toward developing LIGA for high-power applications and there is not any report on a successful THz VED with LIGA fabricated interaction circuit. A fundamental problem with LIGA is the requirement for a synchrotron, which always results in high costs and long development cycles, which is the main reason why the LIGA process is viable in a research environment other than a commercial process.

SU8 photoresist-based UV-LIGA is an effective alternative for LIGA process, it uses ultraviolet radiation instead of X-rays to activate the photoresist. A typical UV-LIGA process only requires several days with a rather low cost, which is attractive for the fabrication of THz interaction circuit. At the time when the traditional machining cannot meet the fabrication requirements of THz devices, the UV-LIGA process was paid more attention. Many UV-LIGA-based techniques, including UV-LIGA/EDM combination, UV-LIGA/CNC combination, multi-step UV-LIGA, embedded monofilament UV-LIGA, were carried out to realize THz interaction circuits. The UV-LIGA fabricated FWG circuits were successfully applied in some lower frequency THz devices, such as W-band TWT, Y-band second harmonic TWT amplifier and G-band TWTs.

Nevertheless, UV-LIGA does not appear to be commonly used technology in THz VEDs at present, mainly due to the specialized equipment, unique skills and procedures required. There are still challenges to be overcome or to be taken seriously:

1. Oxygen-free high-conductivity copper substrate with high parallelism, high flatness and low roughness is necessary.
2. SU-8 photoresist has poor adhesion to copper.
3. Internal stress accumulated in thick photoresist after exposure and thermal treatment causes adhesion failure during or after development.
4. No matter in a multi-step process or a combining process, submicron alignment accuracy is required.
5. Electroforming process without organic additives is required to obtain pure copper structures.
6. Vertical sidewalls with smooth surface are required to minimize power loss.
7. High precision planarization is required to assure parallelism, so as to achieve low variation in waveguide height along the length.
8. Complete removal of SU-8 photoresist from HAR slots is required to obtain final clear structures.
9. UV-LIGA has unsatisfactory compatibility with the aligning and bonding technologies of two halves.

Research on these challenges is in progress, including hydrogen annealing and re-polishing of copper substrate, application of photoresist adhesive, application of UV with different wave length, further process optimization of micro-electroplating, combination of photoresist removal processes, simultaneous fabrication of structure and alignment marks, etc.

With the rapid progress in traditional fabrication techniques, modern micromachining such as micro/nano-CNC can provide the capability required for interaction circuit fabrication. UV-LIGA thereby lost its attractiveness gradually in lower frequency band but held the application potential in 670 GHz and above, because the micro/nano-CNC process still cannot realize satisfying fabrication results due to the restriction of tool size. Although the previous 670 GHz interaction circuit is not applied in actual device, research on interaction circuit fabrication has been carried out for 850 GHz, 1.03 THz and 1.1 THz recently.

DRIE is a process where HAR structures are etched into silicon. These structures can generate molds or serve as a mold themselves for generating metallic structures. DRIE is the most developed quasi-3D microfabrication technology, which is compatible with the advanced silicon process platform. Silicon structures ranging from 2 μm up to 1 mm can be fabricated by DRIE process, with nearly vertical sidewall and smooth surface, and the tolerances for the circuit dimensions are consistent with the use of a photolithographic process.

Multi-layer etching can further improve the verticality of the sidewalls. Thermal oxidation and chemical etching can further improve the surface roughness, assuring that DRIE technology can realize precise structures as expected. In addition, a significant advantage of DRIE approach is the excellent aligning ability. With commercial equipment and well-developed bonding process, two halves of the interaction circuits can be assembled easily with an aligning accuracy within 1 μm.

With the above strengths, the interaction circuits by DRIE process have been applied in VEDs from 95 GHz, 220 GHz up to 670 GHz, 850 GHz and 1.03 THz. It is worth mentioning that the silicon interaction circuit with copper coating is still the only one applied in actual devices of 670 GHz and above.

The weakness of the DRIE structure is also obvious. Silicon has poor thermal conductivity, so the devices are not suitable for high average power operation; it also has poor electric conductivity, so a metal coating layer with excellent electric conductivity is necessary to minimize transmission loss. However, firmly coating of uniform and conformal metal layer with a thickness greater than the skin depth on inner surfaces of HAR structures is challenging.

The coating results by common vacuum evaporating and sputtering are not satisfying: the former is difficult to form continuous coverage at the corners, and the latter is difficult to form uniform film on sidewall and deep bottom. New coating methods, for example high-voltage-sputtering with deep-cavity are proposed recently, but they are all in the early stages of development. Atomic layer deposition (ALD) perhaps is a promising method, as it forms the coating through a multi-step atomic monolayer deposition and can realize a uniform layer in complex inner surfaces. But ALD is a highly time-consuming process, as it requires a great deal of deposition cycles to complete a required metal film thickness.

Furthermore, the robustness of different methods has to be verified by actual devices. In the operation of THz VEDs, the coating films are prone to peel off from the silicon surface due to electron bombardment. This is not a rare situation, because the long electron channel is very thin and the ideal electron transmission in interaction circuit is extremely challenging.

Although its conventional predecessor was once claimed to not be suitable for microfabrication, the new micro/nano-CNC technique with improved machines, tooling, and computer software breaks the restriction. Because of its shorter fabrication time, cheaper cost, and competitive precision, the micro/nano-CNC milling becomes an attractive microfabrication technique for THz interaction circuits.

Micro/nano-CNC milling has been widely used in fabrications for the VEDs of 94 GHz, 220 GHz and 340 GHz. In these applicable frequency ranges, micro/nano-CNC milling has performance comparable to UV-LIGA and DRIE techniques but with a rather simple fabrication flow, due to the elimination of complicated lithographic processes. Although micro/nano-CNC milling has slightly less surface finishing and may not be optimal for mass production, it is efficient for low-volume production in the frequency ranges below 340 GHz. Since THz VEDs are still in their development stages, their mass production is currently not as important as the quality of the devices.

The most obvious limitation of micro/nano-CNC machine for higher frequency application is the size of the cutting tool tip. Although the command resolution of the machine itself is typically of nanometer order, the tool tip’s size restricts the actual obtained resolution. Although the standard end mills with a diameter of 25.4 µm are available, the FWGs with similar critical dimensions are still not realized because of the poor uniformity, surface finish, and corner structure, which can be neglected in lower THz frequency to some extent. It is also is the reason why modified sine waveguides and DSG structures are preferred in the present design of higher THz devices, as both of them can ease the CNC milling fabrication process when compared to FWGs.

Three-dimensional printing is a newly used technology for fabricating THz interaction circuits. A key advantage of this method is the ability to make complex structures in a single operation, eliminating the manufacture of numerous piece parts and assembly operations. Different 3D printing techniques are proposed to realize FWGs, but most of the research is focused on lower frequencies of the W-band and D-band, even though the fabricated FWGs are still not used in actual devices because of their poor dimension tolerance and extremely rough sidewall surface.

In addition, FWGs obtained by direct 3D printing always have porous and loose structure, indicating metal powders with refined size are in urgent need, which are also necessary for the realization of tight dimensional tolerance and improved surface roughness. Three-dimensional polymer FWG structure with metal coating have poor thermal conductivity, and the surface treatment technology should be further developed to achieve uniform metallization in narrow enclosed surfaces. The combination of 3D-printed mold and electroforming process can realize better full metal FWGs. However, the difficulty of the combination is almost the same as the UV-LIGA process, but the fabrication quality is much lower than that of the latter.

Although 3D printing technology is experiencing an enhancement in printing accuracy and surface roughness, the state of the art of the technology can still not meet the requirement of THz interaction circuit. The ideal application of it perhaps is the manufacturing of complex structures with less accuracy requirement, especially those with intricate internal features, which are traditionally machined in multiple blocks and mechanically fit together but can be 3D-printed as one single part.

To make a brief summary, simpler technologies that achieve the required performance will always supplant more complicated ones. The most advanced micro/nano-CNC technique has superiority than UV-LIGA or DRIE in lower THz frequency ranges, such as W-band, D-band, and G-band, because it eliminates the complicated lithographic process but can realize interaction circuits with acceptable dimensional accuracy and surface roughness. Although the micro/nano-CNC technique is not applicable for batch fabrication, it is suitable for commercial production due its lower cost and shorter production time. LIGA is always believed to be suitable only for scientific research, mainly due to its extremely high cost and long processing period. While UV-LIGA and DRIE are likely to dominate the higher frequency THz application above 670 GHz, their microfabrication nature makes them more suitable for the microstructure of interaction circuits. They are applicable for wafer-scale batch fabrication with moderate cost and production cycle, and are suitable for commercial production. The precise aligning and assembling of fabricated metal circuit halves in higher THz ranges are still challenging. Although 3D printing techniques can avoid these challenges naturally, they need significant improvement in printing accuracy and surface roughness for practical applications.

Besides the aligning of interaction circuit halves, the precise alignment and assembly of microfabricated components are also important for THz VEDs. With the help of dowel pins inside the jig, R. Panigrahi et al. successfully aligned and assembled nine layers of copper sheets together to a W-band FWG [176]. This technique can consequently be used in parts alignment and VEDs assembly.

A more effective method is to integrate different functional modules into a single structure and fabricate them a single process. The method does not need the aligning jigs but it simultaneously eliminates the problems of misalignment and the assembly of components. N. Kumar et al. developed a W-band interaction circuit, along with beam adapter region and spent beam collector, all on a single plate. With optimized micro-milling techniques, the integrate structure was realized with a dimensional deviation less than 7 µm and an average surface roughness around 100 nm, which met the requirements of the actual devices well [177]. The developed technique for integration fabrication will have potential application in even higher frequency THz sources.

Recent advances in key technologies drive the progress of VEDs, such as modern simulation-based design methodologies that create smart designs, low-volume flexible manufacturing that reduces production costs and development period, and automatic assembly and inspection that ensure that the assembled devices operate as designed, with maximum yield at the minimum possible cost, etc. Those technologies promise to make VEDs more competitive in both the civilian and military markets [178].

Some emerging technologies, such as nanoimprint lithography and two-photon polymerization, may promote new design ideas of THz interaction circuits and become potential solutions for the current fabrication challenges.

Nanoimprint technology also uses photoresist to assist in pattern transferring, just like in a photolithography process. However, it uses mechanical means instead of visible light or ultraviolet light. The reported processing accuracy of nanoimprint has reached 2 nm, exceeding the resolution achieved by traditional photolithography techniques. The templates of nanoimprint can be reused repeatedly, which can reduce processing costs and effectively shorten processing time. Therefore, nanoimprint technology has the technical advantages of high resolution, easy mass production, low cost, and high consistency, and is considered a promising competitor to photolithography technology.

Two-photon polymerization is a nanoscale 3D printing technology. It focuses femtosecond pulse laser through a high numerical aperture objective lens to form a focal point in uncured photoresist, where the two-photon absorption is triggered and the photoresist is aggregated to form voxels. The nonlinear two-photon absorption mechanism breaks the optical diffraction limitation. Therefore, this technology has the ability to achieve micro- to nano-level features, while also retaining the advantages of traditional 3D printing, including single-step manufacturing without alignment. Two-photon polymerization is widely used in the fields of optics and nanophotonics, and has become a powerful tool for manufacturing miniature optical imaging systems.

Although nanoimprint lithography and two-photon polymerization have not yet been used for microfabrication of THz interaction circuits, their excellent processing capabilities must attract widespread attention and promote experimental research in this field in the near future.

## Figures and Tables

**Table 1:** Skin depths at different frequencies for oxygen-free copper.

**Frequency**	35 GHz	94 GHz	220 GHz	340 GHz	670 GHz	850 GHz	1030 GHz	1100 GHz
**Skin Depth**	350 nm	210 nm	140 nm	110 nm	82 nm	72 nm	66 nm	63 nm

**Table 2:** Some interaction circuits fabricated by nano-CNC machine.

No.	Frequency	Device	Interaction Circuit	Tool Diameter	Inside Ra
1	94 GHz	SBK	CC	457 μm	115 nm
2	220 GHz	SB TWT	DSG	305 μm	74 nm
3	220 GHz	SB TWT	DSG	254 μm	96 nm
4	263 GHz	SB TWT	DSG	254 μm	100 nm
5	346 GHz	SB BWO	DSG	127 μm	>200 nm
6	346 GHz	PB BWO	DCW	76 μm	>200 nm
7	270 GHz	PB TWT	FWG	76 μm	163 nm

**Table 3:** Microfabrication technologies for THz interaction circuits.

Method	Fabrication Complexity	Fabrication Capability	Fabrication Precision	Surface Roughness	Batch Production
LIGA	High	1–1000 μm	≤±1 μm	≤40 nm	Not Applicable
UV-LIGA	High	10–1000 μm	±3 μm	≤50 nm	Applicable
DRIE	High	2–1000 μm	±1 μm	≤20 nm	Applicable
Nano-CNC	Medium	≥25 μm	±3 μm	≤100 nm	Not Applicable
3D printing	Low	≥layer thickness	±1.5 μm	10 μm	Applicable

## Data Availability

Data sharing not applicable.
